# Correlates of early attrition from school sports clubs in male senior high school students: a 2.4-year follow-up study

**DOI:** 10.3389/fspor.2023.1203113

**Published:** 2023-08-16

**Authors:** Takashi Jindo, Naruki Kitano, Koki Nagata, Yuichi Nakahara-Gondoh, Kazuhiro Suzukawa, Toshiya Nagamatsu

**Affiliations:** ^1^ Physical Fitness Research Institute, Meiji Yasuda Life Foundation of Health and Welfare, Hachioji, Tokyo, Japan; ^2^ Division of Art, Music, and Physical Education, Osaka Kyoiku University, Kashiwara, Osaka, Japan; ^3^ Faculty of Health and Sport Sciences, University of Tsukuba, Tsukuba, Ibaraki, Japan; ^4^ Faculty of Integrated Human Studies and Social Sciences, Fukuoka Prefectural University, Tagawa, Fukuoka, Japan; ^5^ Faculty of Sport Science, Nippon Sport Science University, Setagaya, Tokyo, Japan; ^6^ The General Department of Aesthetics, Yamano College of Aesthetics, Hachioji, Tokyo, Japan

**Keywords:** organized sport, bukatsudo, dropout, adolescent, athlete

## Abstract

The correlation between early attrition and school sports clubs has been underexplored. This study aims to clarify the correlates of early attrition from school sports clubs at a private male senior high school in Fukuoka Prefecture, Japan. Of the 928 first-year students, 331 belonging to the school's sports clubs were included in this study. A baseline survey was conducted in May 2017 and a follow-up survey was conducted in October 2019. This study defined early attrition as a student who quit a sports club earlier than April in their third year. Examined correlates for early attrition consisted of biological, intrapersonal, interpersonal, and institutional factors. We used univariate logistic regression analysis, in which early attrition was the response variable and the examined correlates were the explanatory variables, and calculated the odds ratios (OR) and 95% confidence intervals (CI). Overall, 232 students (85.0%) continued to participate in sports clubs after April of their third year, and 41 students (15.0%) experienced early attrition. Statistically significant correlates for early attrition were weight (OR = 0.94, 95% CI = 0.90–0.98), body mass index (OR = 0.84, 95% CI = 0.74–0.97), experience of injury or disability (OR = 0.40, 95% CI = 0.19–0.87), athletic achievement (OR = 0.29, 95% CI = 0.13–0.62), and duration of experience of the sport (OR = 0.99, 95% CI = 0.98–1.00). Our findings suggest that the school officers or family members of students should recognize the possibility of early attrition and provide appropriate support if a student experiences early attrition. The athletic level or norms of school sports club activities may differ among other schools and sports clubs; therefore, it is necessary to examine whether our findings are applicable to other schools and sports clubs.

## 1. Introduction

Sports participation helps adolescents to maintain their physical activity (PA) (1) and has a positive effect on their physical (2) and psychosocial (3) health and development. However, organized community and school sports clubs have high attrition rates (4, 5). Early attrition is associated with poor physical health indicators (4), psychological difficulties, and low quality of life (5, 6). To predict early attrition or develop supportive measures before or after early attrition, the first step is to identify its correlates because there are practical constraints to employing experimental designs in youth sports contexts (7).

Crane and Temple's (8) systematic review revealed five reasons for early attrition: lack of enjoyment, perceptions of competence, social pressure, competing priorities, and physical factors (maturation and injury). Balish et al. (7) found that early attrition was associated with intra- or interpersonal and psychological factors, except for age. Meanwhile, participation (e.g., injury) and institutional (e.g., sport type), and biological factors (i.e., age, anthropometric indicators, and physical fitness) have been insufficiently examined. Further, examinations of the relationship between physical fitness and early attrition (9, 10) and the results of recent previous studies are inconsistent. The existing evidence on the correlates at each factor level is required to understand early attrition.

Adolescent sports participation is important because it would enable them to lead a physically active lifestyle in later life (11). Thus, examining the issue regarding early attrition in adolescents would be beneficial for increasing the number of individuals who have a physically active life in adulthood. Meanwhile, the sports attrition findings are limited to Western countries (7). Major sports settings for adolescent can be classified as school-, community-, and both school- and community-centered, and they can vary across countries (12). While Western countries are mainly categorized as both school- and community-centered, or community-centered, many Asian countries are categorized as school-centered (12). In Japan, the school sports club is a major organized sports setting among Japanese adolescents, and is positioned as an educational activity, not just a sporting activity (12). Approximately 50% of high school students belong to school sports clubs (13), and there is a wide range of athletic levels among the students. It is regarded as the norm for students who belong to a sports club to remain with only one club throughout their school career (14). With such a particular culture, the factors associated with early attrition in Japanese school sports clubs may differ from those in sports organizations in other countries. However, only one longitudinal study of early attrition in Japanese school sports clubs exists, and focuses on motivation and burnout (15). To develop strategies for maintaining sports opportunities and PA for students in Japan, we need to first explore the correlates of early attrition from sports clubs in Japanese adolescents from various perspectives. Subsequently, the identified correlates should be linked to future studies that aim to examine the causal inference or prediction. To consider or interpret the various correlates, a framework of a social ecological model of sports attrition (7), which considers the related factors for behavior on multiple levels, would be useful. The correlates, which may be modifiable or not (7), would be considered as the possible characteristics that are susceptible to early attrition and are helpful to plan preventive or supportive measures regarding early attrition.

Accordingly, the purpose of this study is to explore the correlates of early attrition from various perspectives in accordance with the social ecological model in Japanese male senior high school sports club members with the help of a 2.4-year longitudinal investigation. We hypothesized that the variables related to coping skills or school-life well-being would correlate with early attrition in Japanese school sports clubs in addition to psychological factors, such as perceived competence or athletic social support, because of its particular culture requiring strenuous effort, demanding that students not give up under pressure, and being strongly related to school life.

## 2. Methods

### 2.1. Study design and participants

This study was conducted in a private male senior high school located in Fukuoka Prefecture, Japan. The target school was selected by convenient sampling considering that it had enough students who belonged to sports clubs, and it accepted the longitudinal study design. The school has several strong sports clubs, but there was no specialized sports course. A self-report questionnaire was administered in a physical education class, and the school's annual physical fitness test data were provided by staff. The baseline and follow-up investigations were conducted in May 2017 (first year), May 2018 (second year), May 2019 (third year), and October–December 2019 (about 6 months before graduation).

The inclusion criteria were students who belonged to a school sports club. Of all the first-year students (*n* = 928), we excluded those who were absent from the baseline investigation (*n* = 22) and those who did not consent to using their data for research (*n* = 16). A total of 331 students met the inclusion criteria.

### 2.2. Measures

#### 2.2.1. Early attrition from sports clubs

The follow-up examined patterns of sports club participation throughout high school. Students were asked whether they belonged to a sports club. Those who did not belong to a sports club revealed the year and month that they had quit. The duration of participation in the club was calculated accordingly. For members of a sports club, the duration between the baseline and follow-up investigation (i.e., 30 months) was assigned. For non-members, the duration between the baseline and the time of quitting was assigned. In Japan, students who quit a school sports club earlier than the third year are commonly considered an example of early attrition (14). Therefore, this study defined early attrition as a student who quit a sports club earlier than April of their third year.

#### 2.2.2. Correlates of early attrition

Since only a limited study (15) on the correlates of early attrition in Japanese school sports clubs has been conducted so far, the candidate correlates should be viewed from various perspectives. We set the explanatory variables with reference to previous studies (7, 8). Specifically, in addition to the psychological factors, the inadequately investigated variables, such as injuries or physical disabilities, physical fitness, and athletic achievement, were included as candidate correlates in this study.

As of this moment, the previous systematic reviews that are available have used the social ecological model, which categorizes the variables into biological, intrapersonal, interpersonal, institutional, community, and policy factors (7), or the leisure constraints theory, which categorizes the variables into intrapersonal, interpersonal, and structural factors (8) to interpret the correlates. The current study used the social ecological model to categorize the correlates because detailed categories, such as the biological and intrapersonal factors, seemed to suit this study.

##### 2.2.2.1. Biological factors

Biological factors included height, weight, body mass index (BMI), and physical fitness. Height (using the equipment used for school health checkups) and weight (MC-190; Tanita, Tokyo, Japan) were objectively measured, and the BMI was calculated. Standardized physical fitness testing (16) is conducted annually in all schools in Japan (16, 17) including the target school, and the total score was included in the analysis. Physical fitness testing has been mandated by the government since 1964, and its reliability is established by a test manual (16). The physical fitness test includes the following eight measurements: grip strength (muscle strength), bent-leg sit-up (abdominal muscle strength/endurance), sit-and-reach (flexibility), side step (agility), 50 m sprint (speed), standing long jump (lower body explosive strength), handball throw (upper body explosive strength), and 20 m shuttle run (cardiovascular fitness). The results of each measurement were converted into standardized scores ranging from one to ten using age- and sex-specific Japanese fitness norms, and summed to calculate the total score for the analysis (16). Detailed measurement information for each test (18) and a summary of the physical fitness testing (17) have been described in previous studies.

##### 2.2.2.2. Intrapersonal factors

Intrapersonal factors included injury or disability, athletic achievement, weekly training duration, perceived session rating, duration of the athletic activity, subjective athletic performance, school well-being, and sense of coherence (SOC).

Experience of injury or physical disability that interfered with daily life in the past year was evaluated in the second- and third-year investigations, and the applicable students were also asked about the timing of their experiences. If the timing was prior to the time when they had quit a sports club, they were coded as “Have an experience.”

For athletic achievement, participants were asked about their past year's highest achievement in a competition. The four response options were “Won a prize larger than or equal to a prefecture-level competition,” “Participated in a national-level competition,” “Won a prize at a national-level competition or higher,” and “Won no specific prize in a competition or not applicable.” Students who had any achievements were categorized into “Won a prize larger than or equal to a prefectural-level competition.” The others were categorized as “Won no specific prize or not applicable.”

We assessed the training duration per day (i.e., hours and minutes) and multiplied the training days to calculate the weekly total duration. We measured perceived exertion for usual training sessions using seven response options that ranged from “Nothing at all” to “Extremely hard.” We assessed the duration of experience of the sport (i.e., years and months) to measure students’ participation.

We evaluated subjective athletic performance using an originally-developed item that asked for students’ subjective ratings of their past month's sports performance, regardless of competition results. Responses were provided on an 11-point Likert scale (“Worst performance” = 0, “Best performance” = 10).

We assessed school-life well-being using a 22-item scale developed in previous studies (19). Nine items were on school-life enrichment, six items were on academic performance, and seven items were on career decisions.

We measured SOC using the three-item SOC scale from the University of Tokyo Health Sociology Department (20). This scale evaluates subordinate concepts of SOC (i.e., manageability, meaningfulness, and comprehensibility) using seven response options. The scores range from 3 to 21, with higher scores indicating a higher ability to cope with stress.

##### 2.2.2.3. Interpersonal factors

Interpersonal factors included athletic and general social support. We used the Athletic Social Support Scale (21, 22) to assess participants’ perceived social support related to sports activities. The scale consists of five items: (1) understanding and encouragement, (2) respect and appreciation, (3) direct assistance, (4) provision of information, and (5) sharing of entertainment. The responses range from “Very dissatisfied” (1 point) to “Very satisfied” (5 points). The total score of the five items, ranging from 5 to 25, was used as the social support score. We assessed general social support using the Japanese version of the Social Support Scale (23, 24). The scale consists of 12 items regarding perceived social support from family, significant others, and friends. Seven response options range from “Very strongly disagree” to “Very strongly disagree.” The total score, ranging from 12 to 84, was used for the analysis.

##### 2.2.2.4. Institutional factors

Institutional factors included sport type and athletic team achievement. Sport type was categorized as an individual or team sport and judged by membership at a sports club. Even though some individual sports (e.g., martial arts or racquet sports) are conducted in a team, they were categorized as individual sports. Team athletic achievement was measured using the teams’ past three years’ highest achievement using the same four options as individual achievement.

### 2.3. Statistical analysis

We used SPSS version 27 (IBM, Inc., Armonk, NY, USA) for the data analysis. The statistical significance level was set at *P* < 0.05.

Because 25.7% of the participants and 15 out of 18 analyzed variables had at least one missing data (e.g., the rate of missing data range from 0.6% to 17.5%), these missing data were input in accordance with the missing at random assumption (i.e., the missing value does not depend on the variable itself but on other observed variables). We prepared 100 multiple imputed datasets (25) and analyzed the pooled results from the separately integrated results by applying Rubin's rules (26).

To examine the relationship between each potential correlate and early attrition, we performed a univariate logistic regression analysis and calculated the odds ratio (OR) and 95% confidence interval (CI) for early attrition. Because we aimed to explore the correlates for early attrition and not conduct causal inference or prediction, we reported unadjusted estimates. An OR less than 1 indicates a negative association, whereas an OR greater than 1 indicates a positive association between variables. The further the OR is from 1, the stronger the association.

## 3. Results

**Figure 1:**
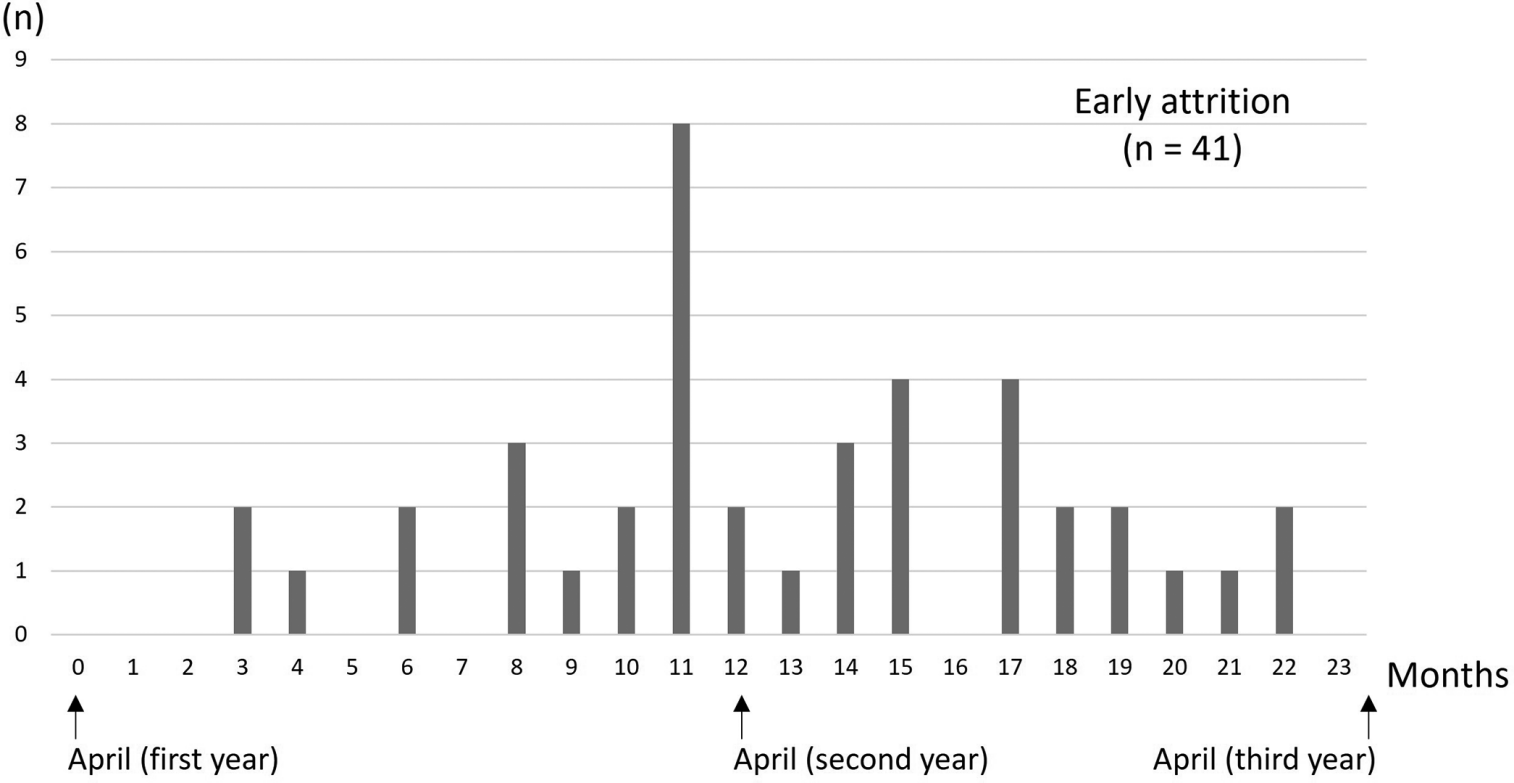
Timing of early attrition.

Overall, 232 students (85.0%) continue to participate in sports clubs after April of their third year, 41 students (15.0%) experience early attrition, and 58 students have missing data. Early attrition occurs during the first year for 19 students and during the second year for 22 students (Figure 1).

Table 1 presents the participants’ characteristics in the imputed and complete data. Experience of injury or disability show the largest missing data rate (*n* = 49, 14.8%) among the explanatory variables.

**Table 1:** Logistic regression analyses results for early attrition (ref.: no early attrition).

Variables	Imputed data (*n* = 331)	Complete data (*n* = 235)	Missing data *n* (%)
Biological factor			
Height, cm (SD)	169.3 (5.7)	169.3 (5.6)	4 (1.2)
Weight, kg (SD)	61.5 (10.4)	61.4 (10.7)	4 (1.2)
Body mass index, kg/m^2^ (SD)	21.4 (3.2)	21.4 (3.2)	4 (1.2)
Physical fitness score, point (SD	61.9 (8.4)	61.5 (8.2)	15 (4.5)
Intrapersonal factor			
Experience of injury or disability			49 (14.8)
No experience, *n* (%)	192 (58.1)	134 (57.0)	
Experience, *n* (%)	139 (41.9)	101 (43.0)	
Athletic achievement			4 (1.2)
Won no specific prize or not applicable, *n* (%)	172 (51.9)	125 (53.2)	
Won a prize larger than or equal to a prefecture level competition, *n* (%)	159 (48.1)	110 (46.8)	
Weekly training duration, hours/week (SD)	19.6 (5.7)	19.3 (5.0)	3 (0.9)
Ratings of perceived exertion for usual training session, point (SD)	5.0 (0.9)	5.0 (0.9)	0 (0.0)
Duration of experience of the sport, years (SD)	6.4 (3.5)	6.5 (3.3)	0 (0.0)
Subjective athletic performance, point (SD)	4.9 (2.0)	4.8 (2.1)	2 (0.6)
School-life well-being			
School-life enrichment, point (SD)	37.6 (6.2)	37.4 (6.2)	16 (4.8)
Academic performance, point (SD)	18.6 (4.1)	18.6 (4.0)	16 (4.8)
Career decision, point (SD)	24.7 (5.1)	24.7 (5.1)	17 (5.1)
Sense of coherence, point (SD)	14.7 (3.5)	14.6 (3.2)	16 (4.8)
Interpersonal factor			
Athletic social support, point (SD)	19.8 (3.0)	19.7 (2.9)	0 (0.0)
General social support, point (SD)	67.0 (13.7)	66.7 (13.2)	16 (4.8)
Institutional factor			
Sport type			4 (1.2)
Individual, *n* (%)	114 (34.3)	77 (32.8)	
Team, *n* (%)	217 (65.7)	158 (67.2)	
Team athletic achievement			3 (0.9)
Won no specific prize or not applicable, *n* (%)	77 (23.1)	58 (24.7)	
Won a prize larger than or equal to a prefecture level competition, *n* (%)	254 (76.9)	177 (75.3)	

SD, standard deviation.

**Figure 2:**
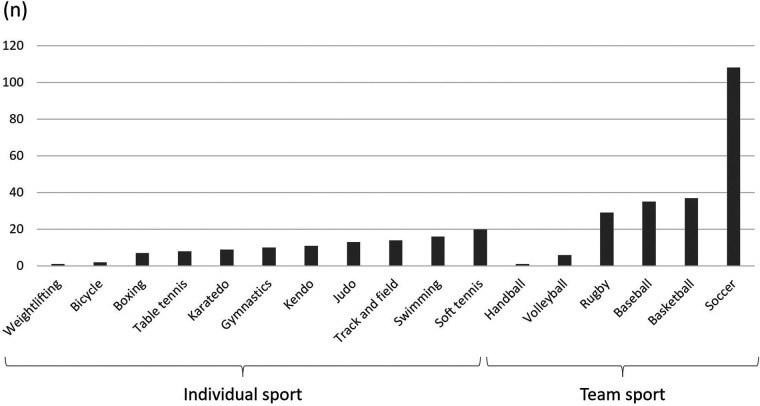
Details of sport type.

Figure 2 shows the students’ sport types. There are 11 individual and 6 team sports clubs. Table 2 shows the results of the logistic regression analyses for early attrition. The results show significant associations between early attrition and weight (OR = 0.94, 95% CI = 0.90–0.98), BMI (OR = 0.84, 95% CI = 0.74–0.97), experience of injury or disability (OR = 0.40, 95% CI = 0.19–0.87), athletic achievement (OR = 0.29, 95% CI = 0.13–0.62), and duration of experience of the sport (OR = 0.99, 95% CI = 0.98–1.00).

**Table 2:** Logistic regression analyses results for early attrition (ref.: no early attrition).

Variables	OR 95% CI
Biological factor	
Height, cm (SD)	0.94 (0.89–1.00)
Weight, kg (SD)	**0.94** ((**0.90–0.98)**
Body mass index, kg/m^2^ (SD)	**0.84** **(**(**0.74–0.97)**
Physical fitness score, point (SD)	0.96 (0.92–1.00)
Intrapersonal factor	
Experience of injury or disability	** **
No experience, *n* (%)	Ref.
Experience, *n* (%)	**0.40** **(**(**0**.**19–0.87)**
Athletic achievement	** **
Won no specific prize or not applicable, *n* (%)	Ref.
Won a prize larger than or equal to a prefecture level competition, *n* (%)	**0.29** **(0.13–0.62)**
Weekly training duration, hours/week (SD)	1.00 (1.00**–**1.00)
Ratings of perceived exertion for usual training session, point (SD)	1.16 (0.78**–**1.72)
Duration of experience of the sport, years (SD)	**0.99** **(0.98–1.00)**
Subjective athletic performance, point (SD)	0.87 (0.75–1.02)
School-life well-being	** **
School-life enrichment, point (SD)	0.98 (0.9398.03)
Academic performance, point (SD)	0.99 (0.91–1.07)
Career decision, point (SD)	1.00 (0.93–1.06)
Sense of coherence, point (SD)	0.98 (0.89–1.09)
Interpersonal factor	
Athletic social support, point (SD)	0.92 (0.82–1.03)
General social support, point (SD)	1.00 (0.98–1.03)
Institutional factor	
Sport type	** **
Individual, *n* (%)	Ref.
Team, *n* (%)	1.04 (0.50–2.14)
Team athletic achievement	
Won no specific prize or not applicable, *n* (%)	Ref.
Won a prize larger than or equal to a prefecture level competition, *n* (%)	0.87 (0.41–1.84)

Univariate analyses were performed. Bold numbers indicate a statistical significance (*p* < 0.05).

OR, odds ratio; CI, confidence interval.

## 4. Discussion

This study examines the association between early attrition and correlates in male senior high school sports club members using a longitudinal design. The results show that injuries or disabilities that affected daily life, individual athletic achievement, and duration of experience of the sport were associated with early attrition. Other variables that were reported as correlates (e.g., subjective athletic performance and social support) did not show statistical significance.

The association between anthropometric and early attrition has been insufficiently examined in a previous review (7). Meanwhile, a cross-sectional study that examined the correlates of early attrition in adolescents who played various sports found that BMI was not associated with early attrition (9). Conversely, a cross-sectional study of adolescent swimmers found that those who had not experienced early attrition were taller (27), and a longitudinal study of adolescent basketball players found that those who continued for 2 years after the baseline were taller and heavier than those who experienced early attrition (10). Based on these findings, anthropometric indicators are associated with early attrition from the viewpoint of the continuation of the same sport, since this is strongly linked to sports performance.

Injury has been proposed as a correlate of early attrition (8). However, while some studies have reported injury as a reason for early attrition (28, 29), others have reported that injury is less important for early attrition (30). The current study's results support the latter finding because the students who did not experience early attrition experienced injury or disability more than those who had no such experiences. Similarly, a previous retrospective descriptive study from Japan reported that 41% of the targeted university students experienced a sport-related injury at high school and more than half of the students with injuries (54%) returned to sports before their complete recovery (31). Although injury or disability can account for early attrition (28, 29), they can also be seen as more frequent events when continuing with sports club activities.

Athletic achievement and duration of sport experience were significantly associated with early attrition. Balish et al.'s (7) review found that adolescent wrestlers’ high athletic achievement in their previous season was associated with low early attrition (30); however, the low study quality and small number of reports renders the evidence insufficient. Subsequent studies have reported that adolescent basketball players with no early attrition had more years of competitive experience than those who experienced early attrition (10). From this lack of conclusive research, the current study originally reports the association between athletic achievement, duration of experience of the sport, and early attrition in a variety of school sports clubs. Students who have high sports achievement or much experience might choose to enter a school with the intention of participating in sports clubs. In addition, it is assumed that there might be some students who try to get admission into colleges or universities through a sports referral. Based on these assumptions, the intention to engage in sports or choose a career path in sports may lead them to avoid early attrition.

Conversely, we did not find statistical significance for other correlates that were confirmed in previous studies from Western countries (7, 8). Specifically, subjective athletic performance, which corresponds to the high-quality correlate of “Perceived competence” (7), should be paid attention. Athletic social support, which includes “Presence of close friendships in sport” and “Coach relationship” as correlates of early attrition (7), was also not significantly associated. This may be because of the lower statistical power due to the small number of early attritions in this study. Meanwhile, a Japanese sports club's specific culture might account for the differences in the correlates. If a club has a norm that views athletic achievement as more important than subjective athletic performance, then that norm might affect the correlates of early attrition. A future investigation that clarifies school sports clubs or students’ norms would be beneficial. Meanwhile, previous studies have reported that athletic social support mitigates burnout (22), and that burnout is associated with early attrition from a school sports club (15). However, it may be the norm for students with lower athletic levels to be encouraged to continue with activities, even though burnout scores tend to worsen as they progress to the next grade (15). Therefore, even if athletic social support affects psychological conditions such as burnout in school sports clubs, it does not affect early attrition due to particular club cultures. With these factors in mind, future studies should consider confirming whether there were students who were not able to stop participating in the sports activity even if they wanted to.

The early attrition rate in this study was 15.0%. Studies from Western countries (that include the same and different sports engagement at the baseline and follow-up) have reported early attrition rates as 33.3% for adolescents aged 10–14 years (5) and 18.5% for adolescents aged 14–17 years (32). A Japanese school sports club study reported 38.0% early attrition by May of the third year (15). Accordingly, there is a difference in the early attrition rate depending on the region, age, and sports organization (participation in the same organization or not) of the target population. Compared to the previous studies, the rate of early attrition in our study's population appears lower, even in the same sports club activities. However, the correlates can be interpreted as characteristics that are susceptible to early attrition among some students. A possible practical application would be school officers or families of students paying attention to students with those characteristics and providing them with appropriate support. Meanwhile, our correlates appear closely related to the athletic level. Preventing early attrition in athletes with low athletic levels may be achieved by treating individual performances as successes rather than comparing them socially (30). Based on these findings, performance-oriented support rather than athletic achievement or social comparison is more important in school sports club activities because of students’ wide range of athletic levels.

This study has several limitations. First, we did not consider some important previously reported factors such as “Competing priorities” and “Intention to dropout” (7, 8). Moreover, the viewpoint of a sports career, such as the intention to get an admission into a college or university through a sports referral, was lacking in this study. These factors may have institutional or cultural differences and may affect early attrition. Further investigations are needed to determine whether these factors are associated with early attrition in various sports settings including school sports clubs, thus leading to a systematic comparison on the institutional and cultural differences. Second, we only evaluated the examined variables (except for injury or disability) at one point in the first year. The status of each factor changes over time (e.g., Tsuchiya, 1998), so it is necessary to consider and analyze such changes. In addition, it should be noted that some variables, such as athletic achievement, training duration, perceived exertion for usual training sessions, and subjective athletic performance, were assessed using non-validated scales. Third, caution is needed when generalizing the results. Our study was conducted in one private male senior high school that had a high athletic level (individual athletic achievement: 48.1%, team athletic achievement: 76.9%). Since the athletic level or norms of school sports club activities may differ among other schools and sports clubs, it is necessary to examine whether our findings are applicable for various schools and sports clubs in the future. The target school was active in competitive sports; therefore, further research is required to investigate whether the findings are applicable to schools with different cultures for sports club activities. In addition, since only male senior high school students could be targeted in this study, future studies should include female, and junior high school or university students who have different athletic levels or belong to a different development stage than the one covered in this study.

## 5. Conclusion

This study reveals that in the target school's sports clubs, early attrition is associated with low weight and BMI, non-experience of injury or disability that affects daily life, low individual athletic achievement, and short duration of experience of the sport. Conversely, other potential correlates proposed in previous studies in Western countries showed no statistical significance. Since school sports clubs have particular cultures, the factors associated with students’ early attrition may also be specific. Our findings suggest that the school officers or families of students should recognize the possibility of early attrition and provide appropriate support if a student experiences early attrition. Future studies that clarify the causal relationship between the correlates and early attrition or its mechanism will help the development of the prevention of early attrition.

## Data Availability

The raw data supporting the conclusions of this article will be made available by the authors, without undue reservation.
